# Hypoglycemia symptoms and awareness of hypoglycemia in type 1 diabetes mellitus: cross-cultural adaptation and validation of the Portuguese version of three questionnaires and evaluation of its risk factors

**DOI:** 10.1186/s13098-020-0521-z

**Published:** 2020-02-14

**Authors:** Paula Stefenon, André Luís Marques da Silveira, Luana Seminotti Giaretta, Cristiane Bauermann Leitão, Andrea Carla Bauer

**Affiliations:** 1 Postgraduation Program in Endocrinology, Faculdade de Medicina, Universidade Federal do Rio Grande do Sul, Porto Alegre, Rio Grande do Sul Brazil; 2 Faculdade de Medicina, Universidade Federal do Rio Grande do Sul, Porto Alegre, Rio Grande do Sul Brazil; 3 Endocrinology Division, Hospital de Clínicas de Porto Alegre, Rua Ramiro Barcelos 2350; Prédio 12; 4° andar, Porto Alegre, 90035-003 Brazil; 4 Nephrology Division, Hospital de Clínicas de Porto Alegre, Porto Alegre, Rio Grande do Sul Brazil

**Keywords:** Hypoglycemia, Diabetes mellitus, Clarke questionnaire, Gold questionnaire, Edinburgh hypoglycemia symptom scale, Cross-cultural adaptation, Validation

## Abstract

**Background:**

To adapt and validate the Clarke and Gold questionnaires and the Edinburgh Hypoglycemia Symptom Scale (EHSS) to Brazilian Portuguese and to determine the prevalence and risk factors associated with impaired awareness of hypoglycemia (IAH) in patients with type 1 diabetes mellitus (T1DM).

**Methods:**

The process of translation, cultural adaptation, and validation of the questionnaires followed the recommendations of the International Society for Pharmacoeconomics and Outcomes Research (ISPOR)-Task Force for Translation and Cultural Adaptation. Patients with T1DM for a minimum of 12 months, aged 18 years or older, and with Brazilian nationality were selected to participate.

**Results:**

A total of 123 patients were enrolled. The Clarke and Gold questionnaires as well as the EHSS exhibited adequate internal consistency, test–retest reliability, and convergent validity. The prevalence of IAH was 38.3% with the Clarke questionnaire and 25.2% with the Gold questionnaire. The prevalence increased with longer duration of diabetes, lower HbA1c, and lower eGFR.

**Conclusions:**

The validation and cross-cultural adaptation of the proposed questionnaires to Brazilian Portuguese were adequate. In this sample of T1DM, the prevalence of IAH was high and associated with a longer duration of T1DM, lower HbA1C and lower eGFR.

## Background

Hypoglycemia is a common side effect associated with the use of exogenous insulin and is considered a major barrier to optimal glycemic control since intensified treatment often increases hypoglycemic events [1]. The International Hypoglycaemia Study Group published, in 2017, a joint position statement on the definition of hypoglycemia, where the glucose concentration below 3.0 mmol/L (54 mg/dL) should be considered sufficiently low to indicate serious hypoglycemia and clinically important [2]. They also state that severe hypoglycemia should not be defined in terms of glucose concentration, but should be considered when there is severe cognitive impairment that requires external assistance for recovery. McCoy et al. showed that patients with severe hypoglycemia had a 3.4-fold higher risk of death compared to those with no or mild hypoglycemia [3]. It is estimated that hypoglycemia is the direct cause of death in 4–10% of patients with type 1 diabetes mellitus (T1DM) [4].

Recognizing warning symptoms of hypoglycemia is critical in order for patient’s self-treatment to avoid severe hypoglycemic episodes. Impaired awareness of hypoglycemia (IAH) is a syndrome in which the ability to detect warning symptoms are reduced or absent. These patients exhibit a nearly sixfold higher frequency of severe hypoglycemia than patients without IAH [5].

Approximately 20–25% of adults with T1DM have IAH [6, 7]. Schopman et al. showed that patients with IAH had a two-fold greater total frequency of hypoglycemic episodes over a 4-week monitoring period and significantly more episodes of asymptomatic hypoglycemia when compared with patients in a normal awareness of hypoglycemia (i.e., non-IAH) group (47% and 14%, respectively) [8].

The National Institute for Health and Care Excellence suggests that diabetic patients should be assessed for hypoglycemia risk using specific questionnaires to identify those at highest risk for severe hypoglycemia [9]. This diagnosis allows re-evaluation of the glycemic target and modifications in treatment to reduce hypoglycemia risk and its complications [10]. Scoring systems such as the Clarke [11] and the Gold [5] questionnaires represent some of the available instruments used to identify patients with IAH. The Edinburgh Hypoglycemia Symptom Scale (EHSS) is another instrument to evaluate symptoms in a typical hypoglycemic episode and helps to characterize patients with IAH [12, 13].

Based on the aforementioned importance of validated instruments to identify IAH and the fact that no such instruments adapted to Brazil are currently available, the aim of this study was to adapt and validate the Clarke and Gold questionnaires and the EHSS to Brazilian Portuguese. Another objective of this study was to determine the prevalence of IAH in patients with T1DM attending a tertiary hospital outpatient clinic by comparing the performance of the three hypoglycemia questionnaires in this population. Finally, we aimed to characterize the clinical and laboratory profiles of such patients as well as the variables associated with IAH.

## Subjects, materials and methods

### Participants

This study was carried out in a diabetes outpatient clinic of a tertiary hospital in Brazil. Eligibility criteria included: aged ≥ 18 years; previous diagnosis of T1DM; disease duration of 12 months at minimum; and Brazilian nationality. The exclusion criteria included developmental disabilities or psychiatric disorders that would pose an obstacle in completing the structured interview. Demographic (age, gender), anthropometric [body mass index (BMI)], clinical characteristics (disease duration, type of treatment, presence of diabetic chronic complications, hypoglycemia frequency) and laboratory [fasting plasma glucose, HbA1c, and creatinine to estimate glomerular filtration rate (eGFR)] data were collected from patients’ electronic records.

### Questionnaires

The Clarke questionnaire [11] is a frequently used instrument to evaluate IAH. It comprises eight questions regarding the patient’s perception of hypoglycemia, the frequency of hypoglycemic episodes, and a subjective estimation of the glycemic threshold for symptom generation. Each answer is classified as either normal awareness (A) or reduced awareness (R). Four or more answers marked as R categorizes a subject as having IAH.

The Gold questionnaire [5] uses a simple 7-point Likert scale (1 = Always aware of hypoglycemia, 7 = Never aware of hypoglycemia) to answer the question “Do you know when your hypoglycemia is starting?”. A score between 4 and 7 is compatible with IAH.

The EHSS [13] is an instrument to evaluate patients’ experiences of symptoms in a typical hypoglycemic episode. It comprises 11 symptoms divided into three domains—neuroglycopenic, autonomic, and malaise, which are evaluated by a 7-point Likert scale “1 = Not at all, 7 = Very severely”.

### Translation and cultural adaptation

The translation and cultural adaptation process followed the recommendations of ISPOR’s Task Force for Translation and Cultural Adaptation [14]. The researchers obtained permission from the main authors of the Clarke and Gold questionnaires and the EHSS to translate, adapt in a cross-cultural manner, and validate the instruments for use in Brazilian Portuguese.

Two independent translators who are native speakers of Brazilian Portuguese and fluent in English performed the initial forward translation of the original instruments into Brazilian Portuguese. This step resulted in two Brazilian Portuguese versions that were subsequently analyzed by an expert committee composed of endocrinologists, linguists, and the two initial translators. The committee identified and corrected, by consensus, any ambiguities and discrepancies of words, sentences, and meanings. This process, called reconciliation, generated the preliminary translated version of each instrument.

The Brazilian Portuguese version was back-translated into English by two independent translators who were native English speakers and fluent in Brazilian Portuguese. The two back-translated versions were then analyzed by the expert committee to identify and correct, by consensus, any discrepancies in format, wording, syntax, meaning, and relevance.

Based on the translations and back-translations obtained in the previous steps, the expert committee analyzed each item (questions and answers) of the questionnaires to define the most appropriate version in terms of conceptual, semantic, and content equivalency (harmonization step), which produced a final pilot version of the Brazilian Portuguese questionnaires.

The next step was to apply a cognitive debriefing test to five outpatients with T1DM, which were of both sexes, varying ages, and different socioeconomic groups in order to ensure the patients were a representative sample of the target population. The principal investigator reviewed the results of the cognitive debriefing to identify translation modifications to facilitate the comprehension of the questionnaires. Proofreading was performed to ensure correction of minor errors prior to the generation of the final version. The whole steps of the translation, cross cultural adaptation, and validation of the questionnaires are shown in Additional file 1.

The final version of the questionnaires was applied to a total of 40 patients.

### Data analysis and psychometric measurements

Demographic and clinical data were described using mean (SD) and frequency (percentage). A t-test was used for continuous variables whereas a Chi square test was used for categorical variables. The relation between two numerical variables was measured by Spearman’s correlation coefficient. To evaluate the reliability and validity of the translated instruments, they were applied in 123 patients (including the initial 40 evaluated). Internal consistency was evaluated by Cronbach’s α (values above 0.7 were considered acceptable). Exploratory factor analysis was performed on the 11 items from the EHSS and the seven questions from the Clarke questionnaire.

Test–retest reliability was calculated to verify intra-observer variability by estimation of the interclass correlation coefficient (ICC) and t-test between times 1 and 2 for the Gold questionnaire and the EHSS (continuous variables) and with Kappa for the Clarke questionnaire (ordinal variables). Since there is no reference test for evaluating IAH, we used the necessity of IV glucose to treat hypoglycemia to assess convergent validity with Spearman’s correlation coefficient. Statistical analyses were performed with SPSS 23.0 and the level of significance was defined as α = 0.05.

## Results

### Translation and cultural adaptation

During the debriefing step, one patient did not understand the meaning of the word “palpitations” in the EHSS; therefore, the expert committee decided to include an explanation of this word in parentheses following the “palpitation” symptom in the questionnaire. Fourteen of the 40 T1DM patients that completed the pre-test questionnaires had an initial difficulty understanding the relevance of the numerical scale on the Likert scale of the EHSS and the Gold questionnaire. After further explanation, all of them were able to answer the questions. The questionnaires averaged < 10 min to complete.

### Descriptive analysis

The clinical and demographic characteristics of the studied patients were as follows: 39.6 ± 11.5 years old; 51.2% male; duration of diabetes 22.37 ± 3.47 years; BMI 24.33 ± 3.47 kg/m [2]; HbA1C 8.42 ± 1.48%; and insulin dose of 0.71 ± 0.22 UI/kg/day. Hypertension was observed in 41.5% of patients, peripheral symmetric polyneuropathy in 27.6%, and retinopathy in 59.3%. 17.1% of patients had an eGFR between 15 and 60 mL/min/1.73 m^2^ and 16.3% had a eGFR lower than 15 mL/min/1.73 m^2^.

### Psychometric measurements

The final versions of the questionnaires were completed by 123 patients (including the initial 40 evaluated) to evaluate the reliability and validity of the instruments. The overall internal consistency of the Clarke questionnaire was demonstrated by Cronbach’s α = 0.804. A separate analysis using one question at a time to evaluate the influence of each item on the internal consistency of the instrument resulted in a Cronbach’s α = < 0.804 for all analyses (Table 1). Inter-rater reliability was assessed by Kappa = 0.712 (95% CI 0.598–0.826).

**Table 1:** Internal consistency of Brazilian Portuguese version of Clarke questionnaire

Question	Cronbach’s alpha	Cronbach’s alpha if item deleted
Total	0.804	
1		0.765
2		0.791
3		0.789
4		0.790
5–6		0.783
7		0.768
8		0.760

Internal consistency of the Gold questionnaire was not performed because this questionnaire comprises only one question. The analysis of the ICC supported the test–retest reliability of the instrument with high agreement (ICC = 0.824; 95% CI 0.651–0.911) and the paired t-test did not show a significant difference in the re-test (mean score = 2.83 vs. 2.91, *p* = 0.734).

Regarding the EHSS, the overall Cronbach’s α was 0.749. When taken one question at a time to evaluate the influence of each item on the internal consistency of the instrument, only the removal of the item “hunger” slightly increased the Cronbach’s α to 0.761. After factorial analyses of the scale we identified four subscales based on the eigenvalues after varimax rotation—neuroglycopenic (confusion, drowsiness, odd behavior, speech difficulty, and incoordination), autonomic (sweating, palpitation, and shaking), malaise (headache and nausea), and hunger (Table 2). Test–retest reliability was performed by subscales, with no difference between means on paired t-test and with ICC (95% CI) as follows: neuroglycopenic = 0.93 (0.72–0.95); autonomic = 0.91 (0.60–0.97); malaise = 0.88 (0.52–0.97) and hunger = 0.75 (0.21–0.96).

**Table 2:** Edinburgh Hypoglycemia Symptom Scale factorial analysis compared with the original study

	Component							
	Neuroglycopenic		Autonomic		Malaise		Hunger	
	A	B	A	B	A	B	A	B
Incoordination	*0.804*	*0.569*	0.201	0.184	− 0.075	0.052		–
Odd Behavior	*0.800*	*0.719*	− 0.052	0.019	0.180	− 0.088		–
Confusion	*0.761*	*0.808*	0.133	0.096	− 0.026	− 0.079		–
Speech Difficulty	*0.737*	*0.699*	0.263	0.024	0.044	0.194		–
Drowsiness	*0.492*	*0.535*	0.093	− 0.092	0.198	0.253		–
Shaking	0.207	0.008	*0.804*	*0.574*	0.000	0.163		–
Palpitation	− 0.004	0.138	*0.719*	*0.443*	0.262	0.418		–
Sweating	0.267	0.156	*0.677*	*0.696*	0.079	0.010		–
Headache	0.011	0.348	0.037	0.138	*0.823*	*0.820*		–
Nausea	0.109	− 0.025	0.194	0.061	*0.766*	*0.547*		–
Hunger	0.007	0.076	0.034	0.724	0.033	0.00	*0.945*	**–**

Eigenvalues A: present study, B: original Study. KMO 0.724 (p < 0.001)

Italics represent values of the four subscales based on the eigenvalues after varimax rotation

**Fig. 1:**
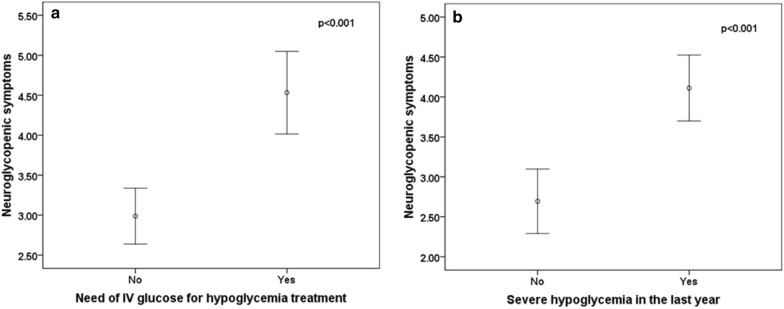
Neuroglycopenic symptoms subscale of the Edinburgh Hypoglycemia Symptom Scale associated with: **a** need of IV glucose for hypoglycemia treatment; **b** presence of severe hypoglycemia

Convergent validity was verified by correlation between the presence of IAH (as diagnosed by the Clarke and Gold questionnaires) and the need for IV glucose for hypoglycemia treatment. Clarke’s correlation was stronger (r = 0.543; *p* = 0.0001) than Gold’s correlation (r = 0.248; *p* = 0.006). In the EHSS, we tested the correlation of the four subscales with the need for IV glucose and with the number of hypoglycemic events in the preceding year. The neuroglycopenic subscale showed a correlation of 0.408 (*p* < 0.001) and 0.410 (*p* < 0.001), respectively (Fig. 1). The Clarke and Gold questionnaires showed a moderate correlation between each other (r = 0.661; *p* < 0001).

The Brazilian Portuguese version of the questionnaires are available at Additional file 2.

### Clinical and laboratory characteristics of patients according to hypoglycemic symptoms

From the 123 T1DM patients evaluated, 55.3% (n = 68) had experienced at least one episode of severe hypoglycemia in the previous 12 months. Of them, 31.7% (n = 39) had received IV glucose to treat hypoglycemia. The prevalence of IAH was 38.3% with the Clarke questionnaire and 25.2% with the Gold questionnaire. The prevalence increased with longer duration of diabetes, from 12.5% in patients with < 10 years of disease to 44% for those with duration > 20 years. It is important to note that 63.4% of the patients have had a diagnosis of diabetes for > 20 years. Table 3 shows the clinical and demographic characteristics of the patients analyzed by the two questionnaires.

**Table 3:** Clinical and demographic characteristics stratified according awareness of hypoglycemia evaluated by the Clarke and Gold questionnaires

	Clarke			Gold		
	Aware (n = 76)	IAH (n = 47)	p	Aware (n = 92)	IAH (n = 31)	p
IAH (%)	61.7	38.3		74.8	25.2	
Sex (% male)	33.1	17.9		42.3	8.9	
Age	38.01 (11.16)	42.09 (11.73)	0.060	39.2 (11.25)	40.68 (12.36)	0.558
BMI	24.19 (3.42)	24.56 (3.59)	0.568	24.17 (3.42)	24.81 (3,60)	0.390
Diabetes duration	20.17 (10.21)	25.91 (9.29)	0.002	21.53 (10.32)	24.84 (9.64)	0.110
Insulin dose	0.72 (0.23)	0.69 (0.20)	0.467	0.71 (0.22)	0.69 (0.20)	0.640
Glycaemia	191.79 (106.53)	166.43 (102.62)	0.196	192.61 (110.8)	150.9 (80.9)	0.028
HbA1C (%)	8.62 (1.52)	8.09 (1.36)	0.047	8.52 (1.49)	8.13 (1.41)	0.199
GFRe (mL/min/1,73 m^2^)	84.05 (36,4)	59.96 (40,68)	0.001	75.82 (39.55)	71.97 (40.65)	0.648
No of hypoglycemia/week	2.2 (1.53)	3.43 (1.97)	0.000	2.38 (1.64)	3.52 (2.03)	0.007
No severe hypoglycemia in 6 m	0.93 (1.82)	3.74 (3.27)	0.000	1.43 (2.13)	3.71 (3.81)	0.003
No severe hypoglycemia in 12 m	2.05 (3.97)	7.28 (6.10)	0.000	3.12 (4.71)	6.81 (6.70)	0.001
Intravenous glucose need	0.11 (0.32)	0.63 (0.48)	0.000	0.25 (0.43)	0.5161 (0.50)	0.006

Variables are described as mean (SD) or percentage (%)

**Fig. 2:**
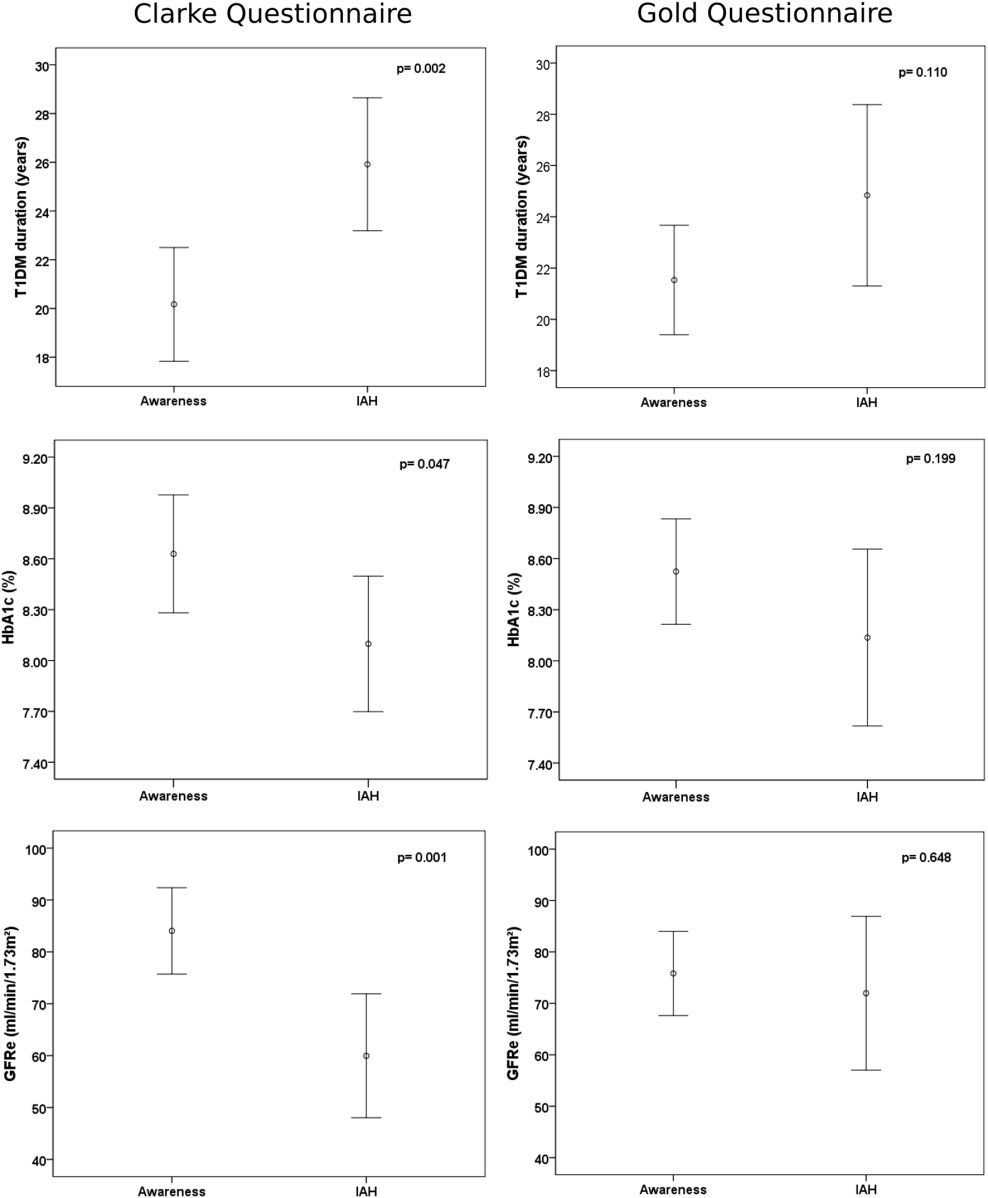
Differences between the Clarke and Gold questionnaires in the ability to detect impaired awareness of hypoglycemia (IAH) according to clinical and laboratory variables, applied in 123 outpatients

Patients classified with IAH by the Clarke questionnaire had a significantly longer duration of diabetes, lower HbA1c, and lower eGFR. No such difference was observed using the Gold questionnaire, as shown in Fig. 2. After multivariate analysis, longer duration of T1DM (*p* = 0.047) and lower eGFR (*p* = 0.014) were shown by the Clarke questionnaire to be independent risk factors for IAH.

## Discussion

This study describes the translation, cross-cultural adaptation, and validation of the Brazilian Portuguese version of two instruments (i.e., the Clarke and Gold questionnaires) to assess IAH in patients diagnosed with T1DM and one instrument (i.e., the EHSS) to characterize hypoglycemic events. The psychometric properties, including internal consistency, reliability, and validity, were assessed. These analyses ensured that the process of adaptation and validation was performed in an appropriate manner and they allowed comprehensible and feasible Brazilian Portuguese questionnaires to be devised in order to identify IAH in T1DM patients in clinical practice. Furthermore, we demonstrate a high prevalence of IAH in our sample (25–38%) and found that a longer diabetes duration and lower eGFR are independent risk factors for this outcome.

In our population, the ability of each questionnaire in detecting IAH differed. The Clarke questionnaire diagnosed IAH in more patients than did the Gold questionnaire, which contrasts with the results found by Geddes et al. of similar prevalence [15]. A possible explanation for this discrepancy relies on the fact that the Clarke questionnaire is a more complete instrument since it evaluates the patient’s exposure to episodes of moderate and severe hypoglycemia and also estimates glycemic thresholds for hypoglycemic responses. The Gold questionnaire, however, is a single-item questionnaire with a 7-point Likert scale, where 1 represents “Always aware of hypoglycemia” and 7 represents “Never aware of hypoglycemia”. During the process of transcultural adaptation of this questionnaire, some patients had difficulties understanding the Likert scale, needing further explanation to be able to answer the question. This fact may, in part, explain the difference in prevalence of IAH between the two questionnaires.

The EHSS is also a 7-point Likert scale (1 = Not at all and 7 = Very severely) where the patients rated the intensity of their symptoms in a typical hypoglycemic episode. Differing from the original study [14] which divided the hypoglycemic symptoms in three subscales (autonomic, neuroglycopenic, and malaise), our data fit more appropriately into four subscales. This decision was based on the factorial analysis of the items, where the “hunger” symptom had a higher score when analyzed in isolation than when analyzed in conjunction with the “autonomic” subscale, as suggested by the original study.

In our study, the IAH prevalence was higher than in other studies [17, 18]. The reasons for this finding might be related to the longer duration of diabetes in our population and the fact that they are treated in a tertiary referral hospital. Previous studies also have shown a higher prevalence of impaired awareness of hypoglycemia with advancing duration of diabetes [16, 17]. Olsen et al. evaluated, by questionnaires of IAH, a cohort of 440 T1DM patients and reported an overall prevalence of IAH of 17% and an increase in the prevalence according to the diabetes duration, from 3% for duration 2–9 years to 28% for duration ≥ 30 years [16]. One possible explanation is that the longer duration of diabetes is associated with lower intensity of autonomic symptoms and a higher prevalence of neuroglycopenic symptoms, which makes it difficult for the patient to recognize hypoglycemia. We also found an association between lower eGFR and the presence of IAH in the Clarke but not the Gold questionnaire.

Chronic kidney disease (CKD) is an independent risk factor for hypoglycemia. The reasons for this include decreased renal clearance of insulin, decreased degradation of insulin in peripheral tissues, reduced renal gluconeogenesis, and prolonged half-life of other medications [18]. In patients on hemodialysis, hypoglycemia is often seen within 24 h after dialysis and occurs as a result of glucose loss during dialysis [19]. However, this effect may also be interpreted as meaning that a high frequency of hypoglycemia is the cause of diabetic kidney disease. This theory, while controversial, states that recurrent hypoglycemia and the consequent release of catecholamines causes arterial stiffness, coagulation abnormalities, and increased inflammatory responses that may lead to renal ischemia and CKD [20].

Patients with predominantly neuroglycopenic symptoms are more likely to have IAH and severe hypoglycemia, as observed in previous studies [21]. We noticed that some patients did not consider low glycemic values as hypoglycemia if they did not have autonomic symptoms. This misunderstanding may contribute to the development of a severe hypoglycemic episode. The patient’s knowledge of the various symptoms of hypoglycemia is crucial to enable its recognition and to reduce episodes of severe hypoglycemia and its consequences [22].

## Conclusion

The process of validation and cross-cultural adaptation of the proposed questionnaires to Brazilian Portuguese was adequate. In this sample of T1DM, the prevalence of IAH was high and associated with longer duration of diabetes and lower eGFR. The high prevalence of IAH among T1DM subjects draws attention to the need for more vigilant care and increased education of these patients in order to reduce morbidity and mortality rates. These questionnaires are of great value to help health care professionals for facilitating the identification of patients at high risk for IAH and to plan interventional strategies to reduce IAH.

## Supplementary information

**Additional file 1.** Translation and cross-cultural adaptation steps of the questionnaires. Summarized table of the process of the translation and cross-cultural adaptation of the questionnaires.

**Additional file 2.** Portuguese version of the questionnaires. Translated, cross-cultural adapted and validated versions of the Portuguese questionnaires: A) Clarke questionnaire, B) Gold questionnaire and, C) Edinburgh Hypoglycemia Symptom Scale.

## Data Availability

The datasets used and/or analysed during the current study are available from the corresponding author on reasonable request.
