# Supplementary material for: Hypoglycemia symptoms and awareness of hypoglycemia in type 1 diabetes mellitus: cross-cultural adaptation and validation of the Portuguese version of three questionnaires and evaluation of its risk factors
Source: Diabetol Metab Syndr. 2020 Feb 14;12:15. doi: 10.1186/s13098-020-0521-z (PMC7023738; doi:10.1186/s13098-020-0521-z)
Supplement: Supplementary file 1 — Additional file 1. Translation and cross-cultural adaptation steps of the questionnaires. Summarized table of the process of the translation and cross-cultural adaptation of the questionnaires. [file 13098_2020_521_MOESM1_ESM.pdf]

CLARKE Questionnaire

Original	Translation 1	Translation 2	Reconciliation	Back Translation	Final
1) Check the category that best describes you: (check one only)	Marque a categoria que melhor descreve você (marque apenas uma):	Marque a categoria que melhor descreve você (assinale apenas uma opção):	Marque a alternativa que melhor descreve você (marque apenas uma):	Select the option which best describes you (one only):	Marque a alternativa que melhor descreve você (marque apenas uma):
2) Have you lost some of the symptoms that used to occur when your blood sugar was low?	Você perdeu alguns dos sintomas que costumavam ocorrer quando seu açúcar no sangue esta baixo?	Você perdeu alguns dos sintomas que costumavam ocorrer quando seu açúcar no sangue esta baixo?	Você deixou de ter alguns dos sintomas que costumava ter quando seu açúcar no sangue estava baixo?	Have you stopped experiencing some of the symptoms you used to when your blood sugar level was low?	Você deixou de ter alguns dos sintomas que costumava sentir quando seu açúcar no sangue estava baixo?
3) In the past six months how often have you had moderate hypoglycemia episodes?(Episodes where you might feel confused, disoriented, or lethargic and were unable to treat yourself)	Nos últimos seis meses, com que frequência você teve episódios de hipoglicemia onde você se sentiu confuso, desorientado ou letárgico e foi incapaz de tratar-se sozinho?	Nos últimos seis meses, com que frequência você teve episódios de hipoglicemia nos quais você se sentiu confuso, desorientado ou letárgico (prostrado) e não conseguiu se tratar sozinho?	Nos últimos seis meses, com que frequência você teve episódios de hipoglicemia em que tenha se sentido confuso, desorientado ou apático e não conseguiu se tratar sozinho	In the last six months, how often have you experienced episodes of hypoglycemia that made you feel confused, disoriented or lethargic and could not self-treat?	Nos últimos seis meses, com que frequência você teve episódios de hipoglicemia (açúcar baixo no sangue) em que tenha se sentido confuso, desorientado ou apático e não conseguiu se tratar sozinho
4) In the past year how often have you had severe hypoglycemic episodes? (Episodes whereyou were unconscious or had a seizure and needed glucagon or intravenous glucose)	No último ano, com que frequência você teve episódios de hipoglicemia onde você esteve inconsciente ou teve convulsões e necessitou glucagon ou glicose intravenosos?	No último ano, com que frequência você teve episódios de hipoglicemia onde você ficou inconsciente ou teve convulsões e precisou de glucagon ou de glicose intravenosa?	No último ano, com que frequência você teve episódios de hipoglicemia em que tenha perdido a consciência (desmaiado) ou tido convulsões, precisando de glicose intravenosa (injeção de glicose) ou glucagon?	In the last year, how often have you experienced episodes of hypoglycemia in which you lost consciousness (fainting) or seizures, requiring intravenous treatment (glucose injection) or glucagon?	No último ano, com que frequência você teve episódios de hipoglicemia (açúcar baixo no sangue) em que tenha perdido a consciência (desmaiado) ou tido convulsões, precisando de glicose intravenosa (injeção de glicose na veia) ou glucagon?
5) How often in the last month have you had readings <70 mg/dl with symptoms?	Com que frequência, no último mês, você teve leituras menores do que 70mg/dl com sintomas?	Com que frequência, no último mês, você teve leituras menores do que 70mg/dl com sintomas?	No último mês, com que frequência você teve medidas de glicose menores do que 70mg/dl com sintomas?	In the last month, how often have your glucose readings been below 70 mg/dl with symptoms?	No último mês, com que frequência você teve medidas de glicose menores do que 70mg/dl com sintomas?
6) How often in the last month have you had readings <70 mg/dl without any symptoms?	Com que frequência, no último mês, você teve leituras menores do que 70mg/dl sem sintomas?	Com que frequência, no último mês, você teve leituras menores do que 70mg/dl sem sintomas?	No último mês, com que frequência você teve medidas de glicose menores que 70mg/dl sem sintomas?	In the last month, how often have your glucose readings been below 70 mg/dl without symptoms?	No último mês, com que frequência você teve medidas de glicose menores que 70mg/dl sem sintomas?
7) How low does your blood sugar need to go before you feel symptoms?	Quão baixo seu açúcar no sangue vai antes que você sinta sintomas?	A quanto desce seu açúcar no sangue antes que você tenha sintomas?	Quão baixo precisa ficar o seu açúcar no sangue para você ter sintomas?	How low does your blood sugar level need to go before you experience symptoms?	Quão baixo precisa ficar o seu açúcar no sangue para você ter sintomas?
8) To what extent can you tell by your symptoms that your blood sugar is low?	Até que ponto você pode dizer pelos seus sintomas que o seu açúcar no sangue está baixo?	Em que extensão você pode dizer, pelos sintomas, que seuee açucar está baixo?	Até que ponto você consegue dizer pelos seus sintomas que o seu açúcar no sangue está baixo?	To what extent can you tell, according to your symptoms, that your blood sugar level is low?	Com que frequência você consegue dizer, pelos seus sintomas, que o seu açúcar no sangue está baixo?

Gold Method (Hypoglycemia Perception Scale)					
**Original**	**Translation 1**	**Translation 2**	**Reconciliation**	**Back Translation**	**Final**
Do you know when your hypos are comencing? Plesase circle one number	Você sabe quando as suas hipoglicemias estão começando? Por favor, circule um número	Você nota quando as suas hipoglicemias estão começando? Por favor, circule um número	Você sabe quando está começando a ficar com hipoglicemia? Por favor, circule um número	Do you perceive when you are starting to get hypoglycemic? Plesase circle one number	Você sabe quando está començando a ficar com hipoglicemia? Por favor, circule um número

Edinburgh Hypoglycemia Scale					
**Original**	**Translation 1**	**Translation 2**	**Reconciliation**	**Back Translation**	**Final**
Please score the extent to which you experience the following symptoms during a typical hypoglycemic episode (circle one value for each symptom):	Por favor, pontue a extensão com a qual você experimenta os seguintes sintomas durante um episódio típico de hipoglicemia (circule um número para cada sintoma)	Por favor, até que ponto você tem os seguintes sintomas durante um típico episódio de hipoglicemia (circule um número para cada sintoma)	Por favor, pontue a intensidade com a qual você tem os seguintes sintomas durante um episódio típico de hipoglicemia (circule um número para cada sintoma).	Please rate the intensity with which you experience the following symptoms during a typical episode of hypoglycemia (circle one value for each symptom).	Por favor, pontue a intensidade com a qual você tem os seguintes sintomas durante um episódio típico de hipoglicemia (circule um número para cada sintoma).
Confusion	Confusão	Confusão	Confusão	Mental confusion	Confusão mental
Sweating	Sudorese	Sudorese	Suor	Sweating	Suor
Drowsiness	Sonolência	Sonolência	Sonolência	Drowsiness	Sonolência
Difficulty speaking	Dificuldade para falar	Dificuldade de falar	Dificuldade para falar	Difficulty to speak	Dificuldade para falar
Pounding heart	Coração batendo (palpitação)	Palpitação	Palpitações	Palpitation	Palpitações (sensação de coração batendo forte)
Hunger	Fome	Fome	Fome	Hunger	Fome
Nausea	Náusea	Náusea	Náusea	Nausea	Náusea
Trembling	Tremor	Tremores	Tremor	Shivering	Tremor
Headache	Cefaléia	Dor de cabeça	Dor de cabeça	Headache	Dor de cabeça
Odd behaviour	Comportamento estranho	Comportamento estranho	Comportamento estranho	Odd behaviour	Comportamento estranho
Incoordination	Falta de coordenação	Incoordenação	Falta de coordenação	Incoordination	Falta de coordenação

Additional file 1: Translation and cross-cultural adaptation steps of the questionnaires.
