# Supplementary material for: Hypoglycemia symptoms and awareness of hypoglycemia in type 1 diabetes mellitus: cross-cultural adaptation and validation of the Portuguese version of three questionnaires and evaluation of its risk factors
Source: Diabetol Metab Syndr. 2020 Feb 14;12:15. doi: 10.1186/s13098-020-0521-z (PMC7023738; doi:10.1186/s13098-020-0521-z)
Supplement: Supplementary file 2 — Additional file 2. Portuguese version of the questionnaires. Translated, cross-cultural adapted and validated versions of the Portuguese questionnaires: A) Clarke questionnaire, B) Gold questionnaire and, C) Edinburgh Hypoglycemia Symptom Scale. [file 13098_2020_521_MOESM2_ESM.pdf]

Additional file 2: Portuguese version of the: **A)** Clarke questionnaire, **B)** Gold questionnaire and, **C)** Edinburgh Hypoglycemia Symptom Scale.

A) Questionário de Clarke

1) Marque a alternativa que melhor descreve você (marque apenas uma):
   ( ) Eu sempre tenho sintomas quando o meu açúcar no sangue está baixo. (PN)
   ( ) Algumas vezes eu tenho sintomas quando o meu açúcar no sangue está baixo. (PR)
   ( ) Eu não tenho mais sintomas quando meu açúcar no sangue está baixo. (PR)
2) Você deixou de ter alguns dos sintomas que costumava sentir quando seu açúcar no sangue estava baixo?
   ( ) Sim (PR)   ( ) Não (PN)
3) Nos últimos seis meses, com que frequência você teve episódios de hipoglicemia (açúcar baixo no sangue) em que tenha se sentido confuso, desorientado ou apático e não conseguiu se tratar sozinho.
   ( ) Nunca (PN)
   ( ) Uma ou duas vezes (PR)
   ( ) Uma vez a cada 2 meses (PR)
   ( ) Uma vez por mês (PR)
   ( ) Mais de uma vez por mês (PR)
4) No último ano, com que frequência você teve episódios de hipoglicemia (açúcar baixo no sangue) em que tenha perdido a consciência (desmaiado) ou tido convulsões, precisando de glicose intravenosa (injeção de glicose na veia) ou glucagon?
   ( ) Nunca (PN)
   ( ) 1 vez (PR)
   ( ) 2 vezes (PR)
   ( ) 3 vezes (PR)
   ( ) 4 vezes (PR)
   ( ) 5 vezes (PR)
   ( ) 6 vezes (PR)
   ( ) 7 vezes (PR)
   ( ) 8 vezes (PR)
   ( ) 9 vezes (PR)
   ( ) 10 vezes (PR)
   ( ) 11 vezes (PR)
   ( ) 12 vezes ou mais (PR)
5) No último mês, com que frequência você teve medidas de glicose menores do que 70mg/dl **com** sintomas?
   ( )Nunca
   ( ) 1 a 3 vezes
   ( ) 1 vez por semana
   ( ) 2 a 3 vezes por semana
   ( ) 4 a 5 vezes por semana
   ( ) Quase diariamente (PR)

6) No último mês, com que frequência você teve medidas de glicose menores que 70mg/dl sem sintomas?
   ( )Nunca
   ( ) 1 a 3 vezes
   ( ) 1 vez por semana
   ( ) 2 a 3 vezes por semana
   ( ) 4 a 5 vezes por semana
   ( ) Quase diariamente (PR)
   (Se resposta da 5< que resposta da 6 = PN; se resposta da 5 < que resposta da 6 = PR)
7) Quão baixo precisa ficar o seu açúcar no sangue para você ter sintomas?
   ( ) 60-69 mg/dL (PN)
   ( ) 50-59 mg/dL (PR)
   ( ) 40-49 mg/dL (PR)
   ( ) Menores de 40 mg/dL (PR)
8) Com que frequência você consegue dizer, pelos seus sintomas, que o seu açúcar no sangue está baixo?
   ( ) Nunca (PR)
   ( ) Raramente (PR)
   ( ) Algumas vezes (PR)
   ( ) Frequentemente (PN)
   ( ) Sempre (PN)

---

3 ou menos respostas PN = percepção normal à hipoglicemia,
4 ou mais resposta PR = percepção reduzida à hipoglicemia.

B) Questionário de Gold

Você sabe quando está começando a ficar com hipoglicemia (açúcar baixo no sangue)?

(Por favor circule um número)

Sempre Percebe						Nunca Percebe
**1**	**2**	**3**	**4**	**5**	**6**	**7**

C) Escala de Hipoglicemia de Edinburgh

Por favor, pontue a intensidade com a qual você tem os seguintes sintomas durante um episódio típico de hipoglicemia (circule um número para cada sintoma).

	Não tenho este sintoma						Tenho este sintoma muito forte
Confusão mental	1	2	3	4	5	6	7
Suor	1	2	3	4	5	6	7
Sonolência	1	2	3	4	5	6	7
Dificuldade para falar	1	2	3	4	5	6	7
Palpitação(sensação do coração batendo forte)	1	2	3	4	5	6	7
Fome	1	2	3	4	5	6	7
Nausea	1	2	3	4	5	6	7
Tremor	1	2	3	4	5	6	7
Dor de cabeça	1	2	3	4	5	6	7
Comportamento estranho	1	2	3	4	5	6	7
Falta de Coordenação	1	2	3	4	5	6	7
